# The Mobile Small RNAs: Important Messengers for Long-Distance Communication in Plants

**DOI:** 10.3389/fpls.2022.928729

**Published:** 2022-06-17

**Authors:** Yan Yan, Byung-Kook Ham

**Affiliations:** ^1^Department of Biochemistry and Biophysics, Texas A&M University, College Station, TX, United States; ^2^Global Institute for Food Security, University of Saskatchewan, Saskatoon, SK, Canada; ^3^Department of Biology, University of Saskatchewan, Saskatoon, SK, Canada

**Keywords:** gene silencing, mobile small RNAs, plasmodesmata, cell-to-cell movement, systemic signaling

## Abstract

Various species of small RNAs (sRNAs), notably microRNAs and small interfering RNAs (siRNAs), have been characterized as the major effectors of RNA interference in plants. Growing evidence supports a model in which sRNAs move, intercellularly, systemically, and between cross-species. These non-coding sRNAs can traffic cell-to-cell through plasmodesmata (PD), in a symplasmic manner, as well as from source to sink tissues, *via* the phloem, to trigger gene silencing in their target cells. Such mobile sRNAs function in non-cell-autonomous communication pathways, to regulate various biological processes, such as plant development, reproduction, and plant defense. In this review, we summarize recent progress supporting the roles of mobile sRNA in plants, and discuss mechanisms of sRNA transport, signal amplification, and the plant’s response, in terms of RNAi activity, within the recipient tissues. We also discuss potential research directions and their likely impact on engineering of crops with traits for achieving food security.

## Introduction

Intercellular signal communication is pivotal for orchestrating plant responses to diverse internal developmental and external environmental cues. Plants utilize a wide array of signal molecules, e.g., ions, phytohormones, proteins/peptides, metabolites, and various forms of RNAs, to mediate in local and systemic responses to various types of input stimuli. In this regard, small RNAs (sRNAs) are important signaling molecules that function in the regulation of both plant developmental processes and abiotic/biotic stress responses. These sRNAs are 21 to 24 nucleotides (nt) in size and can be categorized into three major groups: small interfering RNAs (siRNAs), microRNAs (miRNA), and transfer RNA-derived fragments (tRFs; Hamilton and Baulcombe, 1999; Reinhart et al., 2002; Silhavy et al., 2002; Lee et al., 2009). Dicer or Dicer-like (DCL) 2, 3, and 4 can generate siRNAs from double-stranded RNA (dsRNA) precursors (Molnar et al., 2011), whereas DCL1 produces miRNAs from imperfectly paired hairpin structures of primary miRNA transcripts (Rogers and Chen, 2013). The tRFs were recently identified as a type of sRNA, being mostly generated after cleavage of mature transfer RNAs (tRNA); these tRFs are present in diverse organisms at comparable levels to miRNAs (Cognat et al., 2017; Megel et al., 2019). The sRNAs can be integrated into ARGONAUTE proteins (AGOs), forming the core of RNA-induced silencing complexes (RISC), to mediate either transcriptional gene silencing (TGS) or posttranscriptional gene silencing (PTGS; Brodersen et al., 2008; Voinnet, 2009; Molnar et al., 2011; Cognat et al., 2017).

An expanding level of evidence supports a model in which sRNAs can function, non-cell-autonomously, where they act as mobile signaling agents to control aspects of plant development, defense, and crop yield. In this regard, such non-cell-autonomous sRNAs can traffic, cell to cell, through plasmodesmata (PD), and undergo transport to distal plant organs as well as into plant parasites (Palauqui et al., 1997; Reinhart et al., 2002; Yoo et al., 2004; Molnar et al., 2010; Shahid et al., 2018; Skopelitis et al., 2018). Here, we review recent progress in our understanding of the gene silencing mechanisms mediated by non-cell-autonomous sRNAs in plants.

## Movement of Mobile sRNAs in Plants

The intercellular mobility of sRNAs was first recognized in a study of transgene-triggered gene silencing. Here, grafting studies revealed that a gene silencing signal was transmitted from silenced stocks to non-silenced scions expressing a *uidA* transgene, a glucuronidase (Palauqui et al., 1997). Another experimental system demonstrated that transgenic expression of a green fluorescent protein (GFP) could be silenced, in the upper systemic leaves, through transiently inducing expression of GFP by agro-infiltrating a leaf in the source region of the plant (Voinnet and Baulcombe, 1997). Later, sRNAs were characterized as signaling molecules to induce systemic gene silencing in plants (Hamilton and Baulcombe, 1999; Foster et al., 2002; Klahre et al., 2002; Molnar et al., 2010). For example, when shoots of transgenic *Arabidopsis* plants, expressing an inverted-repeat *GF* construct (part of *GFP*, called IR-GF), were grafted onto GFP-expressing plants, siRNAs of 21, 22, and 24 nt sizes, produced from the IR-GF, were detected in the grafted roots, leading to silencing of the *GFP* transgene (Molnar et al., 2010).

The pathway by which these siRNA signals move, from the shoot to the root, involves PD that mediate the symplasmic exchange of various molecules. Structurally, PD contain a centrally located appressed endoplasmic reticulum (ER), named the desmotubule, and an outer lining provided by the plasma membrane (PM; Robards and Lucas, 1990; Ham and Lucas, 2017). The cytoplasmic space between the ER and PM can allow the intercellular movement of many molecules, where the PD size exclusion limit (SEL), defined by the ER-PM space, plays an important role in controlling such cell-to-cell diffusion of molecules, to establish cellular identities (Lucas et al., 2009; Petit et al., 2020). Next, systemic movement of various molecules involves PD, located along the phloem, which provide symplasmic continuity to connect cells within the whole plant; mature, enucleate, phloem sieve elements communicate with neighboring companion cells through their PD and serve as a conduit for systemic translocation of important macromolecules (Ham and Lucas, 2017).

Over the past two decades, a large body of evidence has been generated that supports the hypothesis that various sRNA traffic, non-cell-autonomously, from cell to cell (Hamilton and Baulcombe, 1999; Hamilton et al., 2002; Juarez et al., 2004; Dunoyer et al., 2005; Kalantidis et al., 2006; Schwab et al., 2006; de Felippes et al., 2011; Skopelitis et al., 2018). Using an artificial reporter system, through which *SULFUR* or *PHYTOENE DESATURASE* inverted-repeat dsRNAs were generated, specifically in phloem companion cells, it was established that sRNAs could move out 10–15 cells beyond the cell in which the gene silencing was initiated (Himber et al., 2003; Kalantidis et al., 2006; Dunoyer et al., 2007; Smith et al., 2007). In some cases, such as in the embryonic hypocotyl, the dilation of the PD aperture allowed for a more extensive cell-to-cell movement of sRNA of up to 35 cells (Kobayashi and Zambryski, 2007).

**Figure 1:**
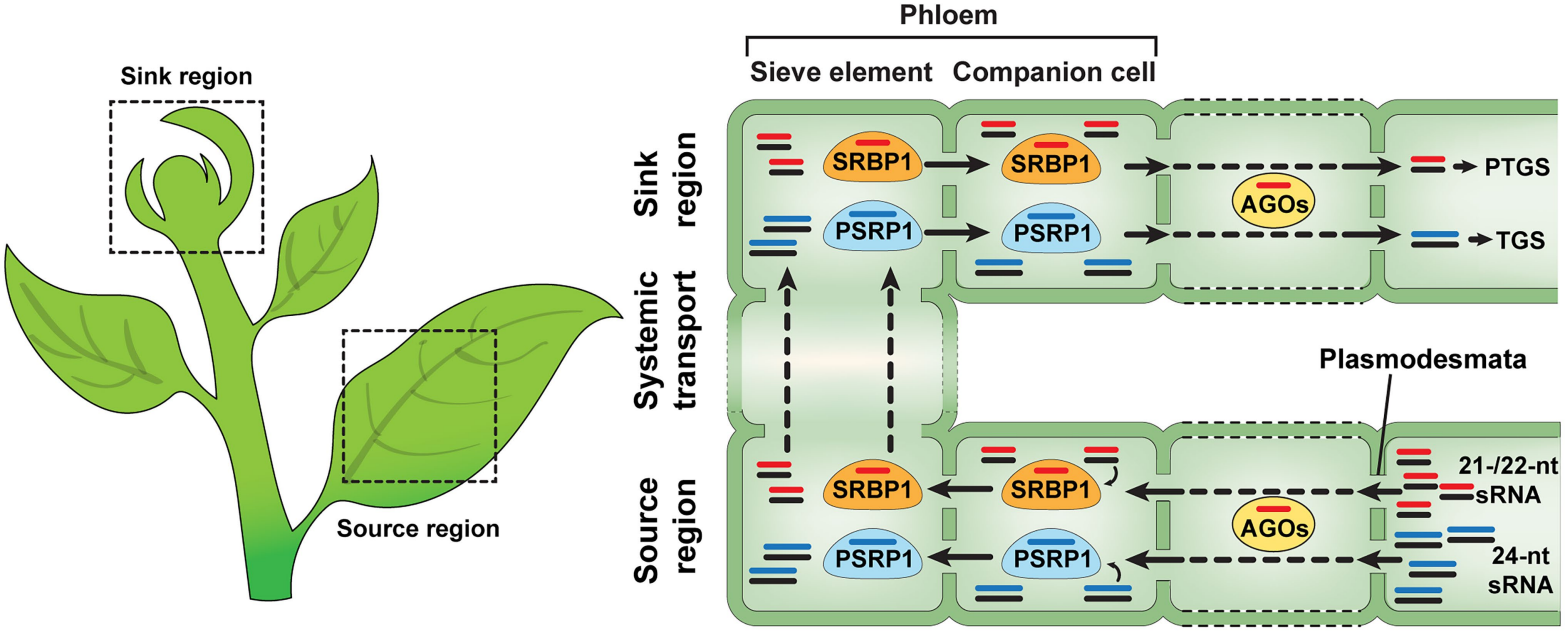
Non-cell-autonomous sRNA trafficking in plants. sRNA duplexes are synthesized in the source regions of plants and move from cell to cell through the plasmodesmata (PD). During sRNA trafficking, mobile sRNAs are consumed by ARGONAUTEs (AGOs), one of the cell-autonomous gene silencing components. PHLEOM SMALL RNA-BINDG PROTEIN1 (PSRP1) and SMALL RNA-BINDIG PROTEIN1 (SRBP1) can mediate non-cell-autonomous transport of ss-sRNAs in plants. It is likely that SRBP1 and PSRP1 prefer to bind 21−/22-nt and 24-nt sRNA, not sRNA duplexes, respectively. All of the 21-, 22- and 24-nt sRNAs can be delivered from source to sink regions, *via* the phloem, and function in transcriptional and posttranscriptional gene silencing within their destined sink tissues (Yoo et al., 2004; Ham et al., 2014; Ham and Lucas, 2017; Skopelitis et al., 2018; Brosnan et al., 2019; Yan et al., 2019; Devers et al., 2020).

Limited information is available on the regulatory mechanisms underlying systemic sRNA-mediated gene silencing. It has been hypothesized that mobile sRNAs move from cells, where they are synthesized, to companion cells for phloem loading in the source tissues (mature leaves) and are unloaded in the target sink tissues after their trafficking along the phloem sieve tube system (Ham and Lucas, 2017). Proteomics analyses and biochemical studies revealed that cucurbit phloem exudate contains a range of RNA-binding proteins, e.g., *Cucurbita maxima* PHLOEM PROTEIN 16, *C. maxima* RNA-BINDING PROTEIN 50, PHLOEM PROTEIN 2, A PHLOEM SMALL RNA-BINDING PROTEIN1 (CmPSRP1), and SMALL RNA-BINDING PROTEIN 1 (SRBP1), that have the capacity to associate with various RNA species and, especially, CmPSRP1 and SRBP1 have been characterized as phloem sRNA-binding proteins (Xoconostle-Cázares et al., 1999; Gómez and Pallás, 2004; Yoo et al., 2004; Ham et al., 2009, 2014; Hu et al., 2016; Yan et al., 2019). CmPPSR1, identified from pumpkin (*C. maxima*) phloem exudate, binds specifically to 24 nt sRNAs and can mediate the cell-to-cell movement of such sRNA (Yoo et al., 2004). This CmPSRP1 forms a ribonucleoprotein (RNP) complex, in the phloem sieve tube system, and a CmPSRP1 KINASE1 (CmPSRPK1) phosphorylates CmPSRP1 to enhance the stability of its RNP complex during phloem-mediated long-distance trafficking of bound sRNAs (Ham et al., 2014). Recently, a new sRNA-binding protein, named SMALL RNA-BINDING PROTEIN 1 (SRBP1), was identified from watermelon, cucumber, and pumpkin phloem exudates, and has close homologs in various plant species (Yan et al., 2019). An *Arabidopsis* homolog of SRBP1, GLYCINE-RICH PROTEIN 7 (AtGRP7), possesses a single-strand sRNA (ss-sRNA)-binding capacity and traffics from cell to cell, suggesting that SRBP1 likely acts as a conserved mediator for non-cell-autonomous gene silencing in many plant species (Yan et al., 2019; Figure 1). Interestingly, another role for AtGRP7 was proposed as a component of the machinery used to transport sRNAs into the apoplast (Karimi et al., 2022). Taken together, these studies indicate that, in *Arabidopsis*, AtGRP7may play a role in the intercellular trafficking of sRNAs, *via* both symplasmic and apoplasmic pathways.

A role for mobile sRNA duplexes, in non-cell-autonomous gene silencing, is supported by numerous findings derived from heterografting studies. First, grafts between a wild-type *Arabidopsis* donor and the *dcl*2,3,4 triple-mutant recipient scion revealed that a long double-stranded precursor RNA is not the mobile form for systemic silencing (Molnar et al., 2010). High-throughput deep sequencing analysis established that a reasonable level of near-complementary miRNA-star (miRNA*) was detected, in grafted recipient tissues, as transmissible sRNAs (Hsieh et al., 2009; Huen et al., 2017, 2018). For example, miR399^*^ could traffic from miR399-overexpressing scions and accumulate within the recipient rootstock, under phosphate (Pi)-deficient conditions (Hsieh et al., 2009). These studies are consistent with the formation of miR399/miR399* duplexes, formed during their phloem-mediated systemic movement. However, as many ss-sRNAs are present in the phloem sieve tube system, and phloem sRNA-binding proteins, such as SRBP1 and PSRP1, appear to function in intercellular transport of ss-sRNAs through the phloem (Yoo et al., 2004; Ham et al., 2014; Yan et al., 2019), it is likely that ss-sRNAs also function as systemic signaling agents.

Insights into the trafficking of sRNA from the vascular tissue into their target cell types were afforded by experiments in which sRNA duplexes were formed by artificial expression, under the control of the vascular stele-specific *SHORTROOT (SHR)* promoter. These sRNA duplexes, bound to a viral silencing suppressor [named *Tomato bushy stunt virus* (TBSV) p19], were detected in root epidermal cells. Interestingly, the depletion of 5′-uridine siRNAs, which associate with AGO1, was observed in the pool of sRNAs detected from the epidermis, suggesting that mobile sRNA duplexes are likely loaded into cell-autonomous AGO1 protein and consumed during their trafficking from the stele to the epidermis (Brosnan et al., 2019; Devers et al., 2020). However, it remains unclear as to whether sRNA duplexes are associated with other protein machineries for their intercellular movement through PD and/or the phloem (Figure 1).

Recently, it was proposed that the intercellular movement of sRNA is precisely regulated at the level of certain cell types. Artificial miRs targeting *GFP* reporter transcripts (miRGFP), expressed under the control of tissue-specific promoters, were transformed into *Arabidopsis* transgenic plants that were constitutively expressing *GFP* in an *RNA-DEPENDENT RNA POLYMERASE6* (*RDR6*)-mutant background, in which tasiRNA production is defective. This system was used to monitor miRGFP movement, through the loss of GFP fluorescent signal, without any silencing effect by newly generated tasiRNA (Skopelitis et al., 2018). Based on the patterns of GFP silencing spread, miRGFP appeared to move, directionally, and selectively, at defined cellular interfaces. Interestingly, the function and movement of miRGFP was restricted within particular domains of the shoot (SAM) and root apical meristems (RAM), but not between other domains, similar with behaviors reported for miR166 and miR394 (Carlsbecker et al., 2010; Knauer et al., 2013).

As free GFP could move across functional domains, this miRGFP movement pattern cannot be explained by simple diffusion of miRGFP from cells where it was produced, due to PD-mediated symplasmic connections. Interestingly, compared with the mobility of miR166, miR394, and miRGFP, miR390 can traffic throughout the SAM and leaf primordia (Skopelitis et al., 2017), suggesting specificity in the regulatory mechanisms for miR transport within the SAM. In *Arabidopsis* hypocotyls, miRGFP, which is produced in phloem companion cells, showed intercellular RNAi within endomermal and other ground tissues; however, miRGFP expressed in ground tissues did not allow GFP silencing in the phloem cells (Skopelitis et al., 2018). Furthermore, miRGFP, derived from shoot-ground tissue, in *Arabidopsis*, had limited entry into the phloem and, therefore, accumulated in the root at a low level, insufficient to induce GFP silencing (Skopelitis et al., 2018). A range of sRNAs, including virus-derived siRNA (vsiRNA), are detected in phloem exudates (Yoo et al., 2004; Buhtz et al., 2008; Varkonyi-Gasic et al., 2010; Rodriguez-Medina et al., 2011; Lewsey et al., 2016; Zhang et al., 2016b), providing support for the hypothesis that entry and/or presence of mobile sRNA in the phloem is a major factor in determining effective transmission of systemic RNAi in plants. Taken together, these findings support the hypothesis that mobility factors, present in PD, act to ensure the polarized movement of specific miRs, and to restrict their transport into phloem companion cells to limit systemic miR trafficking, even though a role for these selective mechanisms, in other species of sRNAs, remains to be established (Skopelitis et al., 2018).

Even though many studies have provided insights into the mechanism of sRNA intercellular movement through PD, it is still unclear what machineries are required for sRNA transport at the PD. As a working model, specific receptors might be present, at each PD orifice, and interact with sRNA-associated proteins for delivery of mobile sRNAs from cell to cell. For ss-sRNA transport through the PD, 21-/22-nt and 24-nt ss-sRNAs are recruited by ss-sRNA-binding proteins, such as SRBP1 and PSRP1 (Yoo et al., 2004; Ham et al., 2014; Yan et al., 2019), and the ss-sRNA-associated proteins can recognize potential PD receptors to first increase PD SEL for intercellular movement of these complexes from CCs to SEs. Other endogenous proteins might be involved in binding sRNA duplexes in CCs, or at PD between CCs and SEs, leading to their non-cell-autonomous trafficking. Future studies, based on RNA-affinity chromatography and/or co-immunoprecipitation approaches, could be employed to identify additional sRNA-binding proteins and PD receptors which can function as mediators to deliver mobile sRNAs between cells. The ss-/ds-sRNAs and sRNA-binding proteins (e.g., SRBP1, PSRP1) would be applicable as “bait” to screen for potential sRNA trasport mediators from phloem exudate and/or plasmodesmata-enriched cell wall fractions (Lee et al., 2003; Yan et al., 2019).

## Mechanism of Gene Silencing by Mobile sRNAs

**Figure 2:**
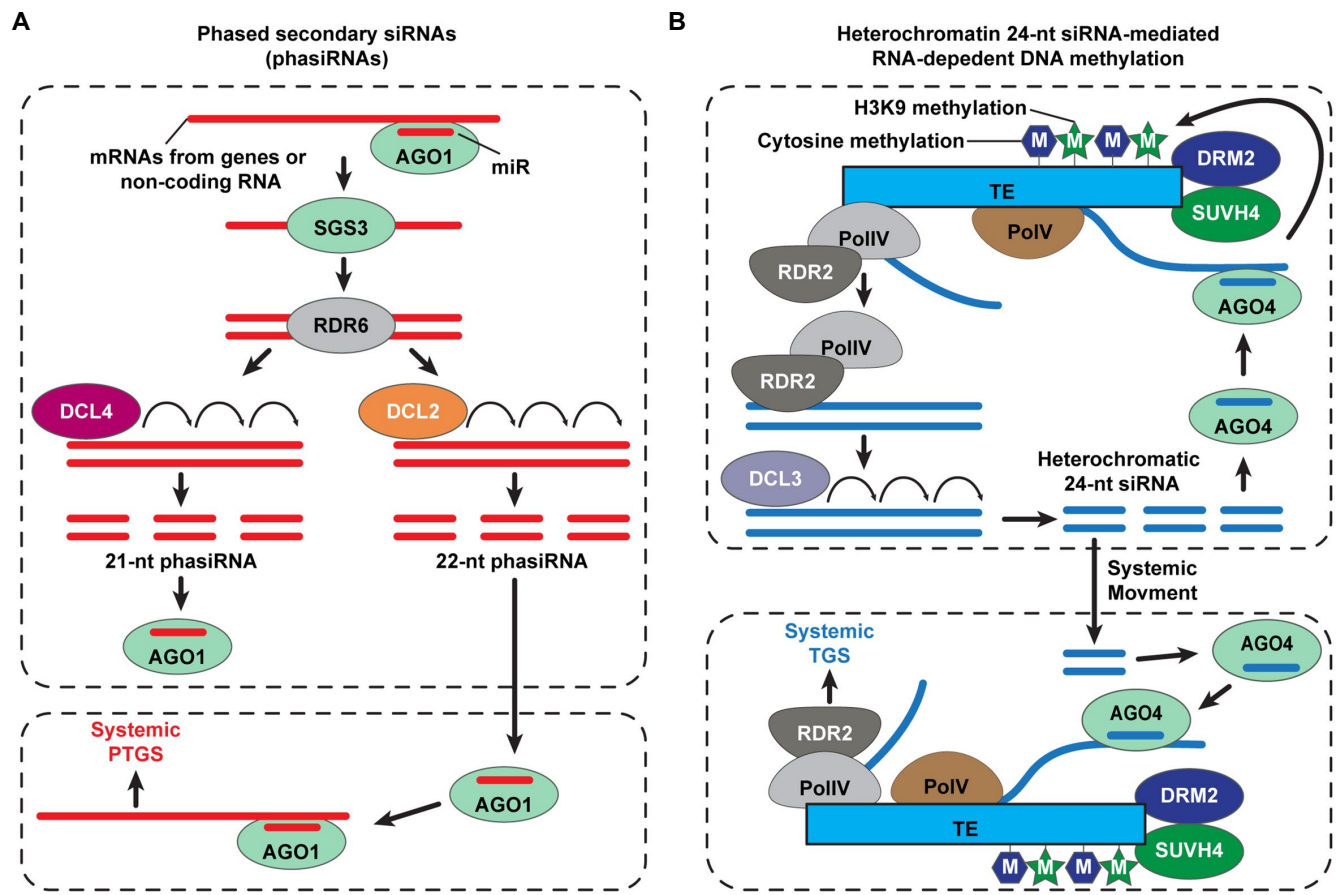
Small interfering RNA (siRNA)-mediated systemic posttranscriptional and transcriptional gene silencing in plants. **(A)** Biosynthesis of phased secondary small interfering RNAs (phasiRNA) for systemic posttranscriptional gene silencing (PTGS) in plants. The ARGONAUTE1 (AGO1) loads a microRNA (miR) and then binds to mRNA transcripts of genes or non-coding RNAs to cleave the target RNAs. Double-stranded RNAs (dsRNAs) are synthesized by RNA-DEPENDENT RNA POLYMERASE6 (RDR6), with dependence on SUPPRESSOR OF GENE SILIENCING3 (SGS3); DICER-LIKE4 (DCL4) and DCL2 cleave long dsRNAs into 21- and 22-nt phasiRNAs, respectively. DCL2-dependent 22-nt phasiRNAs play a role in transitivity to amplify gene silencing signal for non-cell-autonomous systemic PTGS (Chen et al., 2010; Taochy et al., 2017; de Felippes and Waterhouse, 2020; Liu et al., 2020). **(B)** A simplified model of systemic transcriptional gene silencing (TGS), mediated by mobile 24-nt siRNAs. The RNA polymerase IV (Pol IV) initiates the transcription of transposable elements (TEs), and then PolIV-associated RNA-DEPENDENT RNA POLYMERASE 2 (RDR2) generates double-stranded RNAs. The heterochromatin 24-nt siRNAs are cleaved from these double-stranded RNAs by DCL3 and recruited into AGO4. The AGO4-siRNAs target transcripts at homologous TE loci, which are derived from PolV, and recruit *de novo* DNA methyltransferase (DRM2) and/or SU(VAR)3–9 homolog 4 (SUVH4) for methylation of cytosine and histone H3 Lys9 (H3K9), respectively (Chen and Rechavi, 2022); thereby, the TE expression is silenced. Heterochromatic 24-nt siRNAs move and are delivered into the target tissues to trigger systemic TGS in plants (Brosnan et al., 2007; Molnar et al., 2010; Melnyk et al., 2011; Borges and Martienssen, 2015; Martinez et al., 2018; Long et al., 2021; Sigman et al., 2021; Chen and Rechavi, 2022).

A large number of siRNAs are detected in phloem exudates collected from different plant species (Yoo et al., 2004; Buhtz et al., 2008; Kehr and Buhtz, 2008; Varkonyi-Gasic et al., 2010; Rodriguez-Medina et al., 2011; Lewsey et al., 2016). The sRNA amplification, often referred to transitivity, is an essential process for mobile sRNA-mediated gene silencing. A certain transcript, targeted by primary sRNAs, leads to the production of phased secondary siRNAs (phasiRNAs; de Felippes and Waterhouse, 2020). AGO1-associated miR targets a transcript (e.g., protein-coding genes or non-coding RNAs) to form miR-induced silencing complexes (RISC), which then results in double-stranded RNA synthesis, *via* the RDR6 and SUPPRESSOR OF GENE SILENCING 3 (SGS3) complexes (Liu et al., 2020). Next, DCL4 and DCL2 cleave this dsRNA to generate 21- and 22-nt phasiRNAs, respectively, with DCL4 having priority for binding to dsRNA, rather than DCL2 (Borges and Martienssen, 2015). Grafting experiments, using *dcl2* and *dcl4* mutants, revealed that systemic gene silencing was enhanced when using the *dcl4* mutant as the donor tissue; as 22-nt primary siRNA tends to be generated, during transitivity, thus DCL2 plays a role in transitivity and systemic 22-nt phasiRNAs-mediated PTGS (Chen et al., 2010; Taochy et al., 2017; Figure 2A). Together with sRNA abundance in the phloem, the capacity to trigger transitivity might be an important factor to determine mobile sRNAs as functional long-distance RNAi signals.

Transcriptional gene silencing (TGS) requires NUCLEAR RNA POLYMERASE IV (Pol IV), RDR2, and DCL3 as key components for 24-nt siRNA synthesis to guide RNA-directed DNA methylation (RdDM), within the nucleus, and can be triggered by mobile siRNAs in a non-cell-autonomous manner (Borges and Martienssen, 2015). Grafting studies revealed that mobile 24-nt siRNAs trigger RdDM and gene silencing within recipient tissues; mutants for Pol IV, RDR2, and DCL3 were compromised in the perception of mobile 24-nt siRNA-mediated gene silencing signals, which requires RdDM (Brosnan et al., 2007; Molnar et al., 2010; Melnyk et al., 2011; Figure 2B). Although a conflict of evidence exists between different experimental systems, regarding the role of Pol IV, RDR2, and DCL3 in systemic TGS (Dunoyer et al., 2007; Smith et al., 2007), Pol IV and RDR2 likely are common components that function in non-cell-autonomous TGS. Recent studies also reported that Pol IV and RDR2 synthesize not only 24-nt siRNAs but also 21- and 22-nt siRNAs (Martinez et al., 2018; Panda et al., 2020), where the 22-nt siRNAs, generated by Pol IV and RDR2, may serve in the transitivity process for sRNA amplification.

## Functions for Mobile sRNA-Mediated Gene Silencing

### Mobile siRNA for Antiviral Responses, Developmental Patterns, and Reproduction

Virus-derived siRNAs (vsiRNAs) function in the major mechanism for plant antiviral defenses. Such vsiRNAs, generated within virus-infected leaves, move into uninfected plant tissues, where they can systemically initiate antiviral gene silencing (Vance and Vaucheret, 2001). Interestingly, a low level of systemic vsiRNA appears to be sufficient to trigger amplification of vsiRNAs, within the recipient healthy leaves, prior to arrival of the invading virus. In *Arabidopsis*, this has been shown to involve RDRs and DCLs, required in long-distance antiviral responses during virus spread (Garcia-Ruiz et al., 2010; Wang et al., 2010; Qin et al., 2017).

Several studies have reported the role of mobile phasiRNAs in plant developmental patterning. MiR390 targets non-coding *TAS3* RNA to generate phasiRNAs; as a specific type of phasiRNAs, *trans*-acting siRNAs (tasiRNAs) are derived from the miR390-*TAS3* module and serve to regulate *AUXIN RESPONSE FACTOR* (*ARF*) genes (tasiR-ARFs; Montgomery et al., 2008). The mobile tasiR-ARF plays a role in megaspore mother cell (MMC) differentiation within the *Arabidopsis* ovule. Specific expression of *AGO7* and *SGS3* was detected in the nucellus, along the proximal-distal axis, and in epidermal cells of the nucellus, along the medial-lateral axis, respectively. Furthermore, synthesis of these tasiR-ARFs is limited to the epidermis, in which both AGO7 and SGS3 are functional, followed by their trafficking into the hypodermal cells of the nucellar region, which surrounds the MMC, where they act to repress *ARF3* expression for regulation of the MMC differentiation (Su et al., 2017, 2020).

**Figure 3:**
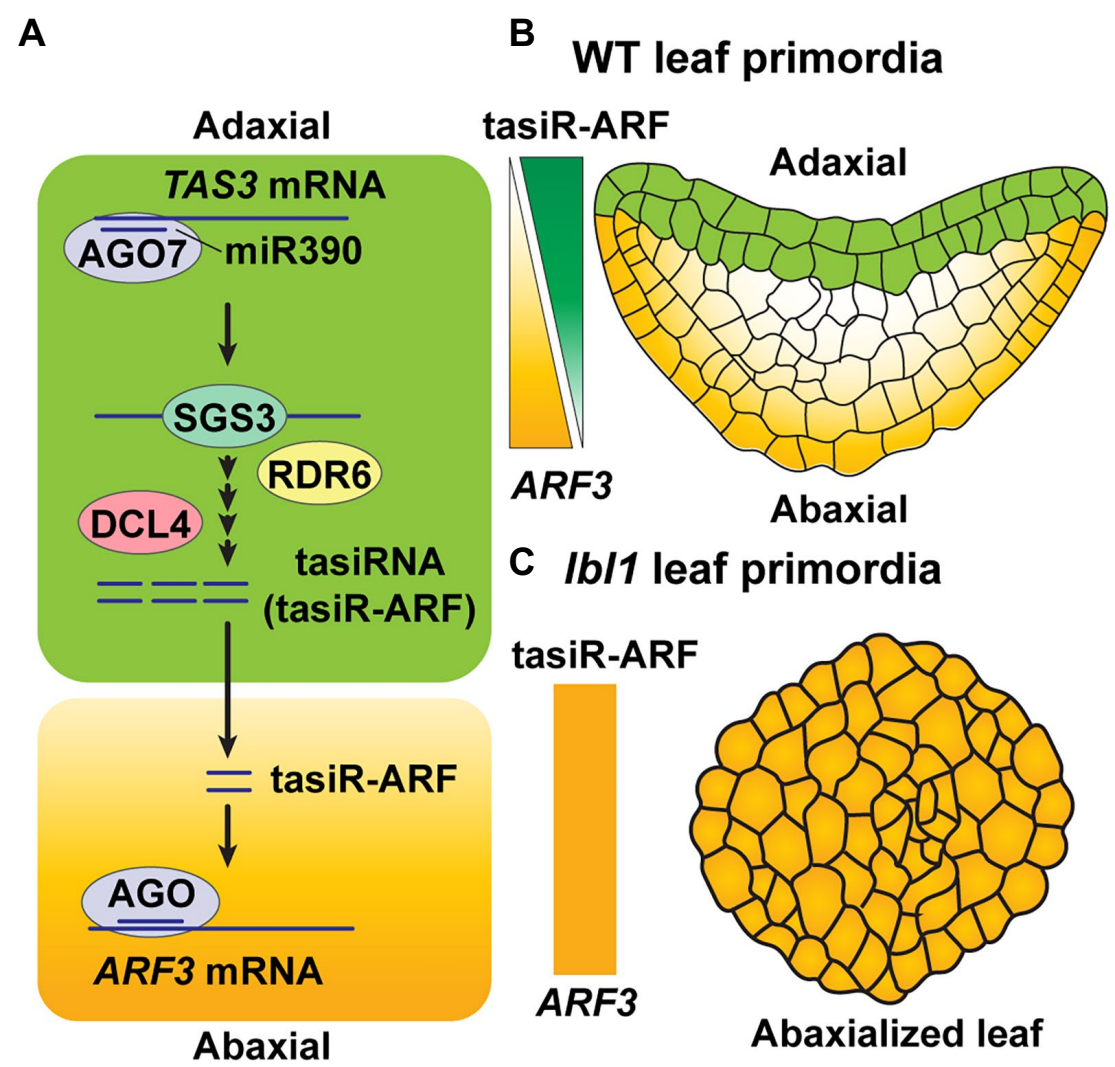
Mobile tasiRNA-mediated leaf patterning. **(A)** Mature miR390 accumulates broadly throughout the leaf in *Arabidopsis*. AGO7, which is specifically expressed on the leaf adaxial side, loads miR390 and then targets *TAS3* transcripts. Production of 21-nt tasiRNAs then occurs through the activities of SGS3, RDR6, and DCL4 (see Figure 1B) in adaxial side of the leaf. The 21-nt tasiRNAs, referred to as tasiR-ARF3, move toward the abaxial area to target *ARF3* transcripts, *in trans*, to repress its expression (Montgomery et al., 2008; Chitwood et al., 2009). **(B)** In *Arabidopsis* wild-type (WT) leaf primordia, tasiR-ARF3 traffics intercellularly, from the top two layers on the adaxial side, where they are synthesized, to form a concentration gradient of tasiR-ARF3 across the leaf; this generates an opposing gradient of *ARF3* accumulation, which establishes leaf polarity (Montgomery et al., 2008; Chitwood et al., 2009). **(C)** LEAFBLADELESS1 (LBL1) is the ortholog of SGS3 in maize. A mutant form of *LBL1*, *lbl1*, disrupts tasiRNA biogenesis on the adaxial layers of leaf primordia, thereby giving rise to an abxialized leaf phenotype in maize (Singh et al., 2018).

Another example for the involvement of tasiR-ARFs in developmental patterning involves leaf primordia in *Arabidopsis*. Here, tasiR-ARFs are synthesized in the two most adaxial cell layers, where AGO7 is expressed, and they subsequently move, from the adaxial to abaxial site, thereby creating a gradient of tasiR-ARF accumulation across the developing leaf (Figure 3A). The end result is a regulation of the *ARF3* target gene for leaf adaxial–abaxial patterning (Chitwood et al., 2009; Figure 3B). Mutation of *LEAFBLADELESS1* (*LBL1*), which encodes the ortholog of SGS3 in maize, leads to abaxialization of leaves, due to a blocking of tasiRNA biogenesis (Singh et al., 2018; Figure 3C), supporting the notion that mobile tasiRNA, specifically produced in adaxial layers, plays an importance role in leaf polarity fate.

The mobility of siRNAs also plays a role in plant reproduction. For example, the 24-nt heterochromatin siRNAs, derived from transposable elements, are synthesized by RDR2 and DCL3 in tapetal nurse cells of *Arabidopsis* anthers, and their trafficking into male meiocytes triggers methylation of targeted transposons and genes in a sequence-dependent manner. Through these mobile, tapetum-derived 24-nt siRNAs, the regulation of gene expression, specifically within the male germline, allows the establishment of inherited RdDM patterns in the anthers, permitting maintenance of genome integrity, across generations (Long et al., 2021).

### Mobile miRs for Plant Development and Stress Responses

Recently, it was proposed that some miRs, which traffic non-cell-autonomously, can function as signaling molecules in control over the formation of developmental patterns and stress responses (Klesen et al., 2020). In support of this notion, heterografting assays performed between soybean and common bean identified shoot-derived miRs in the heterografted recipient roots, consistent with systemic movement of many miRs, *via* the phloem translocation stream, although roles for most mobile miRs still remain to be evaluated (Li et al., 2021).

**Figure 4:**
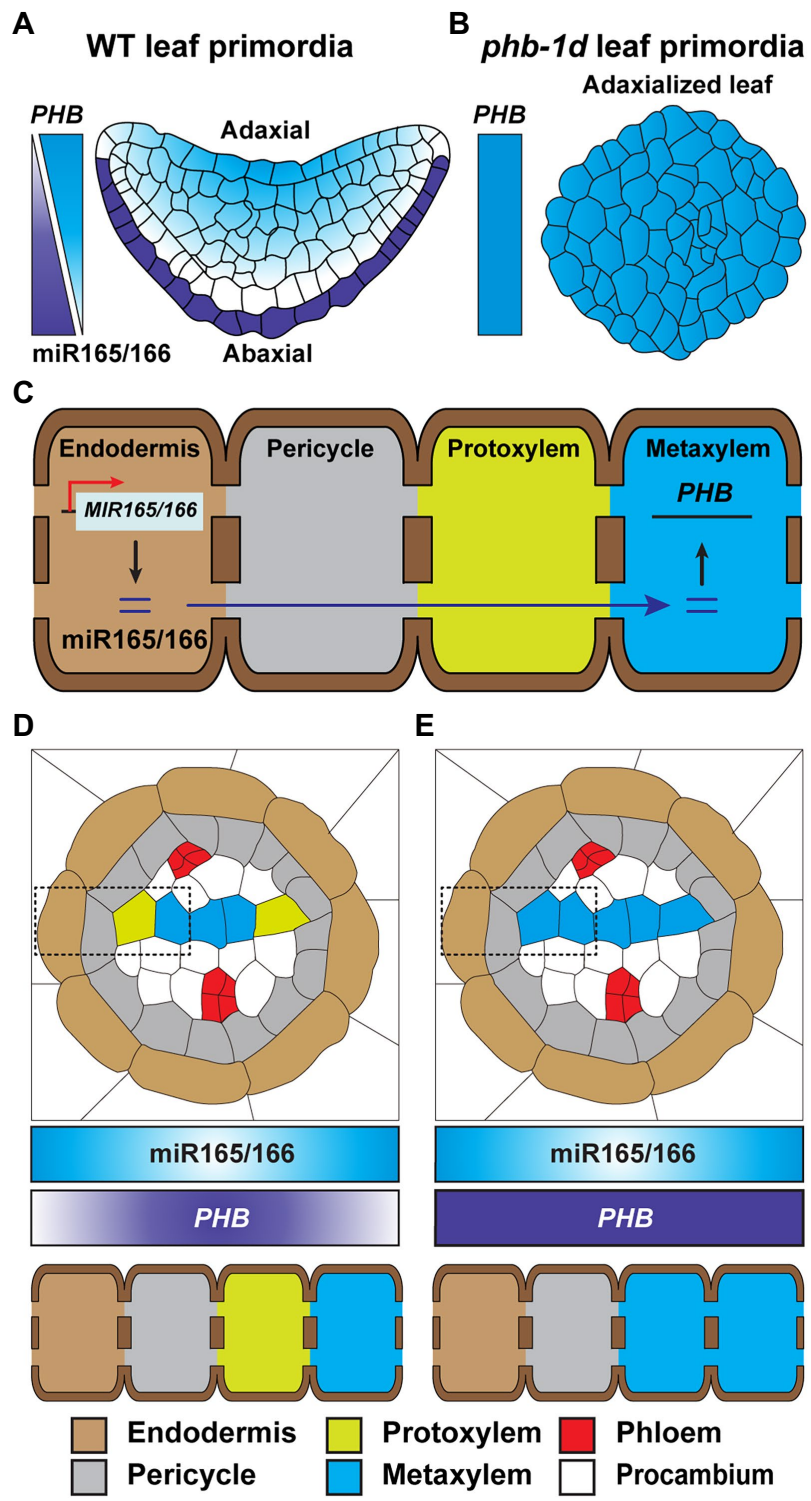
Mobile miR165/166 establishes developmental patterns in leaf and root. **(A)** The biosynthesis of miR165/166 is specifically detected in the abaxial epidermis of *Arabidopsis* WT leaf primordia. The concentration gradient of miR165/166 is generated from the abaxial to adaxial side of the leaf primordia, *via* its intercellular movement, leading to a horizontal level gradient of *PHB* transcript, which is the target gene of miR165/166, from the adaxial to abaxial region, in order to restrict *PHB* expression on the adaxial side and, thereby, confer abaxial–adaxial polarity (Juarez et al., 2004; Yao et al., 2009). **(B)** The *phd-1d* mutant carries a compromised miR165/166-binding site in the *PHB* gene and, thereby, impaired miR-mediated *PHB* silencing. The gain-of-function mutation of *phd-1d* results in a high-level expression of *PHB* in developing leaves, leading to the generation of an adaxialized leaf phenotype (Mcconnell and Barton, 1998; Carlsbecker et al., 2010). **(C)** The mature miR165/166 is strictly produced in the root endodermis. The miR165/166 then moves from the endodermis into the stele in the root, which generates an miR165/166 gradient that decreases toward the root stele. This gradient leads to an inverse gradient in abundance of the miR-targeted *PHB* transcripts. The resultant elevated level of *PHB* expression results in protoxylem and metaxylem formation in the stele (**D**; Carlsbecker et al., 2010; Miyashima et al., 2011; Vatén et al., 2011; Brosnan et al., 2019; Brioudes et al., 2021; Fan et al., 2021). **(E)** Similar to *phd-1d*, shown in **(B)**, a gain-of-function *phd-7d* mutant develops metaxylem at the protoxylem position due to impaired miR165/166-mediated *PHB* gene silencing (Carlsbecker et al., 2010; Miyashima et al., 2011; Fan et al., 2021). In **(D,E)**, endodermis, pericycle, protoxylem, and metaxylem are shown in dotted rectangles.

Spatial analyses of *MIR*-gene promoter activity, mature miR accumulation, and miR-target gene regulation were used to establish an miRNAome and targetome. This resource was employed to understand miR-target gene interaction, at the cell specific level, and led to the identification of a group of non-cell-autonomous miRs within the *Arabidopsis* root and leaf (Brosnan et al., 2019). The miR165/166 has been proposed as a mobile signal involved in cell fate determination. As an example, the expresison of *MIR165* and *MIR166* is strictly detected in the leaf abaxial epidermis; however, the abundance of mature miR165/166 is gradually diminished toward the adaxial side of the leaf. This gradient in miR165/166 serves to regulate the expression of *PHABULOSA (PHB)* that encodes for a class III homeodomain-leucine zipper transcription factor, which then specifies leaf polarity (Figure 4A; Juarez et al., 2004; Yao et al., 2009). The *phb-1d* mutant is a dominant mutant, in which miR165/166-binding sites on the *PHB* gene are disrupted, and shows adaxialized leaf shape (Figure 4B), demonstrating the important role of miR165/166 in leaf polarity (Mallory et al., 2004). In the *Arabidopsis* root, miR165/166 is specifically produced in the endodermis and, subsequently, moves inward into stelar cells inside the endodermis, to establish an abundance gradient in mature miR165/166 that decreases from the endodermis to the stele (Figure 4C; Carlsbecker et al., 2010). Similarly, the abundance of the *PHB* target gradually decreases, from the stele to the endodermis, and functions in the formation of root cell identities (e.g., metaxylem, protoxylem, and pericycle; Miyashima et al., 2011; Figure 4D). Supporting this notion, the gain-of-function *phb-7d* mutant is insensitive to miR165/166-mediated gene silencing; thereby, the *phb-7d* mutant develops metaxylem in the protoxylem position (Figure 4E; Carlsbecker et al., 2010).

A gain-of-function mutation in *CALLOSE SYNTHASE3* (*CALS3*) resulted in callose deposition that then reduced the PD aperture, thereby disrupting the symplasmic movement of miR165/166 in the *Arabidopsis* root tips (Vatén et al., 2011). The distribution of PHB, maintained by the plasma membrane- and PD-localized receptor-like kinases (RLKs) BARELY ANY MERISTEM (BAM)1 and BAM2, likely functions in miR165/166 activity and mobility to ensure proper *Arabidopsis* root xylem patterning (Fan et al., 2021). Recently, a study of HASTY (HST), which encodes EXPORTIN5 required for delivery of precursor miRNAs (pre-miR) from the nucleus into the cytosol in animals, showed that it appears to enable symplasmic movement of miRs, and the *hst* mutant likely suppresses non-cell-autonomous movement of miR165/166 (Brioudes et al., 2021). These findings raise the possibility that BAM1/2 might post-translationally regulate CALS3 activity for PD-mediated trafficking of miR165/166 and that HST plays a role, directly or indirectly, to regulate miR165/166 trafficking from the endodermis to the stele for xylem pole specification in the root.

The miR394 also functions as a mobile signal to suppress the *LEAF CURLING RESPONSIVENESS* (*LCR*) F-box protein mRNA, in a non-cell-autonomous manner. The expression of *MIR394* is limited to the L1 cell layer, during shoot apical meristem (SAM) formation, but mature miR394 is detected within all three stem cell layers (L1, L2, and L3), in order to maintain pluripotency of the SAM by the suppression of LCR in the L3 cell layer (Knauer et al., 2013). Potato underground stem stolon tuberization is also regulated by mobile miRNAs. The miR172 can be transported from *35S::miR172* scions into grafted wild-type stocks, to accelerate tuberization (Martin et al., 2009). On the other hand, miR156 functions as a graft-transmissible signal that suppresses potato tuberization (Bhogale et al., 2014).

In plants, mobile miRs also function as mediators to coordinate nutrient uptake and homeostasis. Phloem exudate contains a range of miRs that exhibit differential levels in response to phosphate (Pi)-starvation stress, suggesting a regulatory role of miRs involved in Pi-stress signaling, *via* phloem-mediated shoot-root communication (Zhang et al., 2016a; Huen et al., 2017, 2018). Under Pi-deficient conditions, miR399 is highly expressed in the vascular tissue of source leaves and its systemic movement to the root was confirmed by grafting studies. Here, when scions overexpressing miR399 are grafted onto wild-type rootstocks, phloem-mobile miR399 is delivered into the root where it targets and downregulate *PHO2*, a ubiquitin-conjugating E2 enzyme, which functions as a negative regulator for root plasma membrane Pi uptake transport (Lin et al., 2008; Zhang et al., 2016b). Another example, in the legume (*Lotus japonicus*), involves miR2111, derived from the shoot, which also moves into the root, where it targets *TOO MUCH LOVE* (*TML*) that functions as a symbiosis suppressor. Thus, miR2111 enhances nodule numbers in the legume root system, suggesting that mobile miR2111 serves as systemic signal to regulate nodulation for nitrogen fixation (Tsikou et al., 2018; Okuma et al., 2020).

## Inter-species sRNA Crosstalk

It has been proposed that sRNA, derived from plants, can move into different species and be used as a biocontrol method. For example, dsRNAs, synthesized from transgenes, can traffic into parasitic plants, fungi, and water mold, to trigger gene silencing (Koch and Kogel, 2014). Although it is a challenge to distinguish sRNA pools in different organisms and evaluate activity of gene silencing, mediated by inter-species sRNA, recent studies have provided evidence for cross-species RNAi processes.

In the parasitic plant, *Cuscuta campestris*, an haustorium, serves as a feeding structure to extract water and nutrients from its plant host. During the infection process, the haustorium accumulates 22-nt miRs, transported into the *Arabidopsis* host plants, which target host mRNAs in a trans-acting manner to trigger phasiRNA synthesis for regulating the expression of host genes (Shahid et al., 2018; Johnson et al., 2019). On the other hand, host-derived sRNAs can be transferred into parasite cells to also regulate gene expression, a process termed host-induced gene silencing (HIGS). As an example, in dodder (*Cuscuta pentagona*), delivery of plant host-derived *SHOOT MERISTEMLESS-like* (STM) sRNA can silence dodder *STM*, which then disrupts its growth (Alakonya et al., 2012). In tomato, generation of siRNAs, derived from dsRNA transient expression, using *Tobacco rattle virus*, or stable expression in transgenic tomato lines, reduced targeted gene transcripts within parasitic plants, resulting in significant decreases in parasitic plant growth on the tomato host (Bandaranayake and Yoder, 2013; Dubey et al., 2017).

Recent studies have also provided evidence of sRNA transport between host plants and fungal pathogens. For example, miRs can be transported from cotton plants into *Verticillium dahliae*; cotton miR159 and miR166 were detected in fungal hyphae collected from infected cotton, and they targeted fungal virulence genes, *ISOTRICHODERMIN C-15 HYDROXYLASE* (*Hic-15*) and *CA^2+^-DEPENDENT CYSTEINE PROTEASE* (*Clp-1*), respectively (Zhang et al., 2016a). The phasiRNAs, derived from *PENTATRICOPEPTIDE REPEAT-CONTAINING PROTEIN* (*PPR*) transcripts, move from the *Arabidopsis* hosts into *Phytophthora capsica* leading to RNAi in *Phytophthora* to neutralize virulence (Hou et al., 2019). Plants and *Rhizobia* bacteria interaction can be mediated by tRNA-derived small RNA fragments (tRFs) to regulate nodulation in the legume; *Rhizobia* generates 21-nt tRFs to regulate soybean host genes involved in root hair development and nodule formation (Ren et al., 2019).

**Figure 5:**
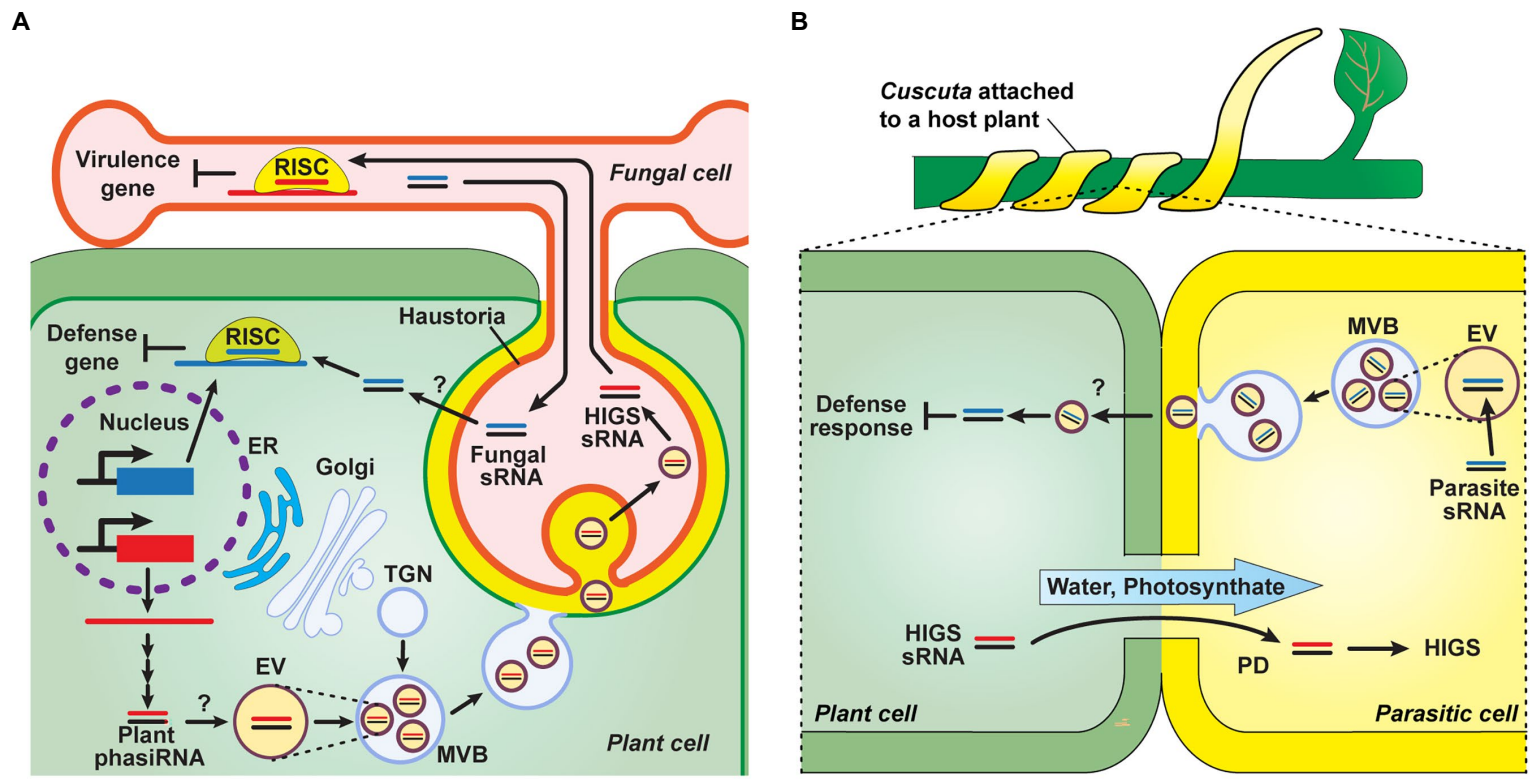
Genetic communication between plants and other organisms, *via* mobile sRNAs. **(A)** Plant-microbe sRNA-mediated interactions. Plants generate phasiRNAs, which are contained in EVs, are delivered into fungal pathogens, e.g., *Phytophthora*, to target pathogen virulence genes to downregulate their expression as a means for plant defense. These EVs are formed within the lumen of MVBs that then deliver these EVs to pathogen cells, at the host-pathogen interface (haustoria), *via* MVB fusion to the host plasma membrane and subsequent uptake into the cytoplasm of pathogen cells. Internalized sRNAs are incorporated into RNA-induced silencing complexes (RISC) to activate host-induced gene silencing (HIGS), which can suppress the virulence of plant pathogens. As a counter-defense strategy, pathogen-derived sRNA might be secreted into the host plant, *via* potential endocytic pathways, and recruited in host RISC to silence plant defense genes. TGN, *trans*-Golgi network; MVB, multivesicular body; and EV, extracellular vesicle (Zhang et al., 2016a; Cai et al., 2018; Baldrich et al., 2019; Hou et al., 2019; Ren et al., 2019; Karimi et al., 2022). **(B)** Hypothetical model of sRNA trafficking between host plants and the parasite *Cuscuta*. Similar to sRNA transport between plants and fungi **(A)**, sRNAs may be loaded to EVs within parasitic plants and exported into the apoplast. Apoplastic movement of sRNAs from parasitic to host plants then mediates in silencing of host defense responses, at the infection sites. The formation of PD, between cells of the host and parasite, establish a symplasmic path for movement of both photosynthate and host sRNA into the parasitic plant (Alakonya et al., 2012; Bandaranayake and Yoder, 2013; Dubey et al., 2017; Shahid et al., 2018; Johnson et al., 2019; He et al., 2021).

Studies on the mechanisms underlying sRNA transport between plants and other organisms remain as a work in progress. Recent studies provided insights into the potential delivery mechanism for inter-species sRNA crosstalk, which can be mediated by extracellular vesicles (EVs). In *Arabidopsis*, purified EVs contained sRNA species of mainly tiny 10- to 17-nt sizes and appeared to act as carriers to mediate the transfer of sRNA, such as miRs and tasiRNAs, between host plants and *Botrytis cinerea* (Cai et al., 2018; Baldrich et al., 2019; Figure 5A). These sRNAs were associated with several RNA-binding proteins potentially involved in EV loading, as they were detected both outside and inside of these EVs (He et al., 2021; Karimi et al., 2022). A range of tasiRNAs, which can target fungal genes, were detected in the apoplastic fluid, and protease trypsin and RNase A treatment assays revealed that these sRNAs, detected in the apoplast, appeared to be more located outside of the EVs (He et al., 2021; Karimi et al., 2022). Although the pathway of sRNA transport from the apoplast to other organisms is unclear, it is plausible that sRNAs are loaded into EVs, which can serve as exosomes to export their sRNA cargo to apoplast, and then a potential mechanism, e.g., endocytosis, might function in EV uptake into the recipient cells for delivery of sRNAs-associated EVs into different organisms. In parasitic plants, the growing ends of *Cuscuta* hyphae are filled with EVs (Vaughn, 2003); therefore, it is hypothesized that EVs are involved in sRNA transport from the parasite to its host plant. Once PD are established between the plant host and its parasite, symplasmic transport of host-derived sRNAs can occur into the parasite to downregulate genes involved in parasitism (Figure 5B).

It has been proposed that plant sRNAs can be transmitted to insects and nematodes (Fishilevich et al., 2016; Mongelli and Saleh, 2016; Zhu et al., 2017). Next-generation sequencing analyses detected plant-derived miRNAs in insects (Wang et al., 2017; Thompson et al., 2019; Zhang et al., 2019) and, in a moth hemolymph (*Plutella xylostella*), two plant-derived miRNAs likely target *BASIC JUVENILE HORMONE-SUPPRESSIBLE PROTEIN 1* and *POLYPHENOL OXIDASE SUBUNIT 2* genes (Zhang et al., 2019), suggesting that these miRNAs, ingested by insects, can be delivered through the gut and regulate target genes in these pests. The dsRNAs, or hairpin RNAs, appear to be major delivery forms of sRNA for this cross-kingdom RNAi, and the length of these dsRNAs, at least 60 nt, is an important factor for effective RNAi between plants and insects (Bally et al., 2020). As agricultural applications, using this knowledge of cross-kingdom RNAi, amiRNAs can be developed to engineer crop plants for the control of agricultural pests. For instance, amiRNA was expressed in tobacco and likely silenced the *HaAce1* gene to disrupt the growth of *Helicoverpa armigera* (Saini et al., 2018). Similarly, *Chilo suppressalis* (Rice striped stem borer, RSB), which when fed on transgenic rice expressing amiRNA to regulate *Spook* and *Ecdysone receptor* genes in RSB, showed developmental defects and high mortality; thus, this rice transgenic amiRNA can confer resistance to RSB (He et al., 2019). Clearly, cross-kingdom RNAi technology has important potential as a next-generation pest control strategy to protect crop plants from insect attack.

## Conclusion and Future Prospects

In this review, we discuss the roles of mobile sRNAs play as important signaling messengers that have pivotal roles in a wide range of biological processes. Although numerous studies have provided evidence for the mobility of sRNA in plants, some fundamental mechanisms remain to be elucidated. First, the molecular determinants that impart the capacity for selective transport of siRNA-protein complexes for directional sRNA trafficking through PD need to be identified. Second, as only a limited number of sRNA-binding proteins, involved in non-cell-autonomous sRNA movement, have been identified and characterized, studies are needed to expand this list of central players in local and systemic gene silence. In addition, studies are also needed to expand our understanding of the mechanisms underlying RNA-binding protein-mediated sRNA transport, specifically in terms of sRNA unloading and the activation/regulation of RNAi processes within the recipient cells. Third, during the process of cross-species sRNA trafficking, specific machineries are likely involved in conferring compatibility between different organisms, and this aspect requires attention. Studying these open questions will have broad impacts on our understanding of the evolution and function of sRNA plant signaling systems.

Considerable evidence offers support for the notion that mobile sRNAs regulate a broad range of processes, including plant stem cell niche maintenance, leaf and root development, potato tuberization, mineral nutrient uptake, and abiotic stress responses in crop plants (Aung et al., 2006; Knauer et al., 2013; Bhogale et al., 2014; Cai et al., 2018; Shahid et al., 2018; Tsikou et al., 2018; Ren et al., 2019; Yang et al., 2019; Fan et al., 2021). In addition, mobile sRNAs can also function as biocontrol agents in crop protection against insects, bacteria, fungi, viruses, and parasitic weeds. Advancing our understanding on these important processes will open new avenues for the engineering of novel mobile sRNA-mediated gene regulatory pathways to produce elite crops with traits for achieving food quality and security.

## Author Contributions

YY wrote the first draft of the manuscript. YY and B-KH contributed to the article and finalized the submitted manuscript. All authors contributed to the article and approved the submitted version.

## Funding

Financial support for this article was provided by National Science Foundation (1906060 to YY) and Natural Sciences and Engineering Research Council of Canada (#RGPIN-2019-04421 to B-KH).

## Conflict of Interest

The authors declare that the research was conducted in the absence of any commercial or financial relationships that could be construed as a potential conflict of interest.

## Publisher’s Note

All claims expressed in this article are solely those of the authors and do not necessarily represent those of their affiliated organizations, or those of the publisher, the editors and the reviewers. Any product that may be evaluated in this article, or claim that may be made by its manufacturer, is not guaranteed or endorsed by the publisher.
